# Supplementary material for: MetaRibo-Seq measures translation in microbiomes
Source: Nat Commun. 2020 Jun 29;11:3268. doi: 10.1038/s41467-020-17081-z (PMC7324362; doi:10.1038/s41467-020-17081-z)
Supplement: Supplementary file 10 — Supplementary Data 7 [file 41467_2020_17081_MOESM10_ESM.zip › File2/Confidence_High_Taxonomy/415848_out.krona.html]

Javascript must be enabled to view this page.

members
magnitude
magnitudeUnassigned
count
unassigned
taxon
rank

415848\_out

460

460
2
superkingdom

976
460
phylum

class
200643
460

460
171549
order

1
1853231
family

genus
283168
1

1
28118

SRS078665\_contig\_number\_96
species

family
815
457

816
22

SRS011239\_contig\_number\_23591SRS013638\_contig\_number\_863SRS013951\_contig\_number\_36368SRS014287\_contig\_number\_2387SRS015782\_contig\_number\_27224SRS019910\_contig\_number\_10134SRS020233\_contig\_number\_2516SRS049773\_contig\_number\_18996SRS049995\_contig\_number\_11759SRS052697\_contig\_number\_42199SRS053356\_contig\_number\_48329SRS076976\_contig\_number\_20112SRS077454\_contig\_number\_16056SRS1041137\_contig\_number\_32210SRS142923\_contig\_number\_20638SRS144297\_contig\_number\_1650SRS144506\_contig\_number\_48356SRS146812\_contig\_number\_55438SRS147346\_contig\_number\_20814SRS147557\_contig\_number\_27408SRS148721\_contig\_number\_50431SRS149325\_contig\_number\_14308
457
genus

species

SRS019161\_contig\_number\_28350SRS052697\_contig\_number\_13754SRS147346\_contig\_number\_38802
3
2292939

species
266
821

SRS011084\_contig\_number\_24211SRS011302\_contig\_number\_25505SRS011302\_contig\_number\_30119SRS011405\_contig\_number\_942SRS012902\_contig\_number\_23SRS013158\_contig\_number\_171SRS013521\_contig\_number\_179SRS013965\_contig\_number\_15209SRS014313\_contig\_number\_2037SRS014459\_contig\_number\_21776SRS014534\_contig\_number\_118SRS014736\_contig\_number\_7090SRS014855\_contig\_number\_5006SRS014979\_contig\_number\_26327SRS015065\_contig\_number\_5129SRS015190\_contig\_number\_11184SRS015217\_contig\_number\_28044SRS015369\_contig\_number\_1360SRS015486\_contig\_number\_358SRS015578\_contig\_number\_27467SRS015579\_contig\_number\_14125SRS015663\_contig\_number\_29638SRS015854\_contig\_number\_17807SRS015960\_contig\_number\_12190SRS016056\_contig\_number\_1342SRS016132\_contig\_number\_141SRS016203\_contig\_number\_2700SRS016495\_contig\_number\_12370SRS016753\_contig\_number\_1814SRS016840\_contig\_number\_103SRS016897\_contig\_number\_156SRS017103\_contig\_number\_33290SRS017191\_contig\_number\_17035SRS017247\_contig\_number\_8322SRS017307\_contig\_number\_16786SRS017375\_contig\_number\_51SRS017433\_contig\_number\_13474SRS017745\_contig\_number\_685SRS017821\_contig\_number\_12259SRS017859\_contig\_number\_1262SRS018351\_contig\_number\_17013SRS018427\_contig\_number\_5083SRS018623\_contig\_number\_5220SRS018623\_contig\_number\_12646SRS018817\_contig\_number\_17154SRS018836\_contig\_number\_26130SRS018888\_contig\_number\_9297SRS018936\_contig\_number\_3231SRS019030\_contig\_number\_989SRS019068\_contig\_number\_69360SRS019178\_contig\_number\_747SRS019267\_contig\_number\_1701SRS019381\_contig\_number\_3984SRS019397\_contig\_number\_983SRS019445\_contig\_number\_3811SRS019496\_contig\_number\_10597SRS019693\_contig\_number\_7823SRS019808\_contig\_number\_19619SRS019968\_contig\_number\_6413SRS020328\_contig\_number\_9730SRS020394\_contig\_number\_9221SRS020508\_contig\_number\_8SRS021219\_contig\_number\_9329SRS021484\_contig\_number\_6963SRS022071\_contig\_number\_28147SRS022524\_contig\_number\_11329SRS023526\_contig\_number\_27882SRS023829\_contig\_number\_150SRS023971\_contig\_number\_1488SRS024009\_contig\_number\_8664SRS024075\_contig\_number\_574SRS024625\_contig\_number\_12005SRS042284\_contig\_number\_1440SRS043411\_contig\_number\_2924SRS043701\_contig\_number\_5160SRS043768\_contig\_number\_12477SRS044535\_contig\_number\_19744SRS045004\_contig\_number\_2949SRS045645\_contig\_number\_3984SRS045739\_contig\_number\_3085SRS046068\_contig\_number\_2096SRS046369\_contig\_number\_8820SRS046502\_contig\_number\_14033SRS046810\_contig\_number\_contig-100\_75.76SRS048164\_contig\_number\_27692SRS048870\_contig\_number\_2674SRS049402\_contig\_number\_1191SRS049446\_contig\_number\_2115SRS049712\_contig\_number\_13304SRS049900\_contig\_number\_9758SRS049959\_contig\_number\_28510SRS049995\_contig\_number\_14292SRS050026\_contig\_number\_3965SRS050422\_contig\_number\_16046SRS050520\_contig\_number\_1154SRS050752\_contig\_number\_10892SRS050801\_contig\_number\_2866SRS050941\_contig\_number\_1297SRS050974\_contig\_number\_1808SRS050998\_contig\_number\_9148SRS051031\_contig\_number\_2461SRS051610\_contig\_number\_186SRS051882\_contig\_number\_13716SRS052027\_contig\_number\_5016SRS052576\_contig\_number\_141SRS053087\_contig\_number\_2569SRS053398\_contig\_number\_24129SRS054059\_contig\_number\_4616SRS054905\_contig\_number\_16369SRS055966\_contig\_number\_239SRS056259\_contig\_number\_11271SRS056273\_contig\_number\_16849SRS056537\_contig\_number\_52305SRS057478\_contig\_number\_772SRS057480\_contig\_number\_10332SRS058268\_contig\_number\_6SRS062427\_contig\_number\_8368SRS062654\_contig\_number\_21177SRS062701\_contig\_number\_4144SRS063127\_contig\_number\_434SRS063370\_contig\_number\_1257SRS063489\_contig\_number\_2856SRS063518\_contig\_number\_29654SRS063813\_contig\_number\_4977SRS063928\_contig\_number\_62SRS064757\_contig\_number\_21999SRS064973\_contig\_number\_94SRS065176\_contig\_number\_4678SRS074586\_contig\_number\_4789SRS074670\_contig\_number\_2990SRS075078\_contig\_number\_16721SRS075398\_contig\_number\_446SRS075716\_contig\_number\_330SRS075773\_contig\_number\_12424SRS075821\_contig\_number\_19818SRS075984\_contig\_number\_19821SRS076756\_contig\_number\_11229SRS076876\_contig\_number\_159SRS077194\_contig\_number\_22633SRS077231\_contig\_number\_1081SRS077294\_contig\_number\_2816SRS077392\_contig\_number\_18263SRS077502\_contig\_number\_3498SRS077552\_contig\_number\_12995SRS077589\_contig\_number\_2472SRS077641\_contig\_number\_22463SRS077849\_contig\_number\_27536SRS077991\_contig\_number\_contig-100\_3.23910SRS078242\_contig\_number\_3223SRS097889\_contig\_number\_18363SRS097958\_contig\_number\_18109SRS098061\_contig\_number\_8251SRS098073\_contig\_number\_11684SRS098571\_contig\_number\_28904SRS098644\_contig\_number\_14411SRS098827\_contig\_number\_1559SRS100021\_contig\_number\_2199SRS100907\_contig\_number\_8655SRS101376\_contig\_number\_12500SRS103987\_contig\_number\_2545SRS104036\_contig\_number\_2817SRS104084\_contig\_number\_5425SRS104084\_contig\_number\_10744SRS1041031\_contig\_number\_13501SRS1041032\_contig\_number\_12440SRS1041036\_contig\_number\_230SRS1041037\_contig\_number\_4585SRS1041090\_contig\_number\_2611SRS1041091\_contig\_number\_17691SRS1041092\_contig\_number\_333SRS1041116\_contig\_number\_7154SRS1041118\_contig\_number\_904SRS1041129\_contig\_number\_6677SRS1041133\_contig\_number\_24660SRS1041137\_contig\_number\_23340SRS1041138\_contig\_number\_9184SRS1041139\_contig\_number\_2730SRS1041140\_contig\_number\_10784SRS1041141\_contig\_number\_9872SRS1041142\_contig\_number\_53SRS1041142\_contig\_number\_2005SRS1041143\_contig\_number\_2947SRS1041144\_contig\_number\_135SRS1041147\_contig\_number\_1025SRS1041157\_contig\_number\_5374SRS104327\_contig\_number\_1860SRS104327\_contig\_number\_6860SRS104400\_contig\_number\_13164SRS104485\_contig\_number\_137SRS104693\_contig\_number\_3339SRS104912\_contig\_number\_3099SRS104975\_contig\_number\_14005SRS105082\_contig\_number\_9866SRS105153\_contig\_number\_19567SRS1054716\_contig\_number\_10252SRS1054928\_contig\_number\_1024SRS1055038\_contig\_number\_1024SRS1055049\_contig\_number\_1435SRS1055056\_contig\_number\_3645SRS1055069\_contig\_number\_10121SRS1055076\_contig\_number\_10236SRS1055083\_contig\_number\_2192SRS1055083\_contig\_number\_8383SRS1055095\_contig\_number\_774SRS140513\_contig\_number\_11730SRS142542\_contig\_number\_6303SRS142781\_contig\_number\_3010SRS142890\_contig\_number\_38140SRS142980\_contig\_number\_2488SRS143070\_contig\_number\_12957SRS143148\_contig\_number\_2909SRS143181\_contig\_number\_13798SRS143342\_contig\_number\_77SRS143417\_contig\_number\_2918SRS143780\_contig\_number\_11325SRS143876\_contig\_number\_12596SRS143895\_contig\_number\_20154SRS143991\_contig\_number\_22945SRS144183\_contig\_number\_20541SRS144362\_contig\_number\_23003SRS144537\_contig\_number\_9760SRS144603\_contig\_number\_2092SRS144714\_contig\_number\_13915SRS144753\_contig\_number\_11694SRS145308\_contig\_number\_519SRS145497\_contig\_number\_12127SRS146764\_contig\_number\_29928SRS146813\_contig\_number\_6838SRS147022\_contig\_number\_926SRS147271\_contig\_number\_464SRS147425\_contig\_number\_7252SRS147614\_contig\_number\_1638SRS147653\_contig\_number\_5352SRS147766\_contig\_number\_371SRS147977\_contig\_number\_383SRS148091\_contig\_number\_10217SRS148196\_contig\_number\_5527SRS148253\_contig\_number\_3775SRS148424\_contig\_number\_17979SRS148784\_contig\_number\_14435SRS148970\_contig\_number\_560SRS149181\_contig\_number\_17768SRS149244\_contig\_number\_603SRS149325\_contig\_number\_contig-100\_287.110421SRS892882\_contig\_number\_contig-100\_6.96003SRS893170\_contig\_number\_11057SRS893172\_contig\_number\_952SRS893187\_contig\_number\_4549SRS893252\_contig\_number\_10870SRS893253\_contig\_number\_322SRS893259\_contig\_number\_694SRS893270\_contig\_number\_24028SRS893285\_contig\_number\_302SRS893292\_contig\_number\_2795SRS893295\_contig\_number\_9843SRS893327\_contig\_number\_1301SRS893341\_contig\_number\_1504SRS893342\_contig\_number\_7629SRS893358\_contig\_number\_10267SRS893366\_contig\_number\_1592SRS893369\_contig\_number\_8260SRS893373\_contig\_number\_22235SRS893378\_contig\_number\_10051SRS893380\_contig\_number\_790SRS893382\_contig\_number\_452SRS971275\_contig\_number\_14389

20
2292941

SRS011134\_contig\_number\_29240SRS013521\_contig\_number\_4606SRS017375\_contig\_number\_13SRS019685\_contig\_number\_6953SRS045004\_contig\_number\_408SRS049995\_contig\_number\_8684SRS052027\_contig\_number\_858SRS054352\_contig\_number\_17302SRS076756\_contig\_number\_880SRS1041036\_contig\_number\_1041SRS1041091\_contig\_number\_20234SRS1041095\_contig\_number\_1815SRS1041147\_contig\_number\_369SRS1055095\_contig\_number\_843SRS143181\_contig\_number\_10563SRS146764\_contig\_number\_18175SRS147088\_contig\_number\_340SRS893172\_contig\_number\_726SRS893187\_contig\_number\_16958SRS893369\_contig\_number\_1177
species

species
46
2292944

SRS011302\_contig\_number\_7133SRS012969\_contig\_number\_21453SRS013382\_contig\_number\_514SRS014459\_contig\_number\_24245SRS015065\_contig\_number\_4177SRS015369\_contig\_number\_7266SRS015486\_contig\_number\_495SRS016056\_contig\_number\_3976SRS017433\_contig\_number\_3132SRS018427\_contig\_number\_4177SRS018836\_contig\_number\_16772SRS020328\_contig\_number\_27429SRS023526\_contig\_number\_21864SRS042966\_contig\_number\_17263SRS045195\_contig\_number\_1278SRS049900\_contig\_number\_48SRS050801\_contig\_number\_2053SRS050941\_contig\_number\_460SRS062701\_contig\_number\_6626SRS063370\_contig\_number\_612SRS064557\_contig\_number\_16413SRS064645\_contig\_number\_32SRS076976\_contig\_number\_12018SRS077294\_contig\_number\_4125SRS077552\_contig\_number\_2066SRS077849\_contig\_number\_23316SRS078242\_contig\_number\_15777SRS078665\_contig\_number\_568SRS100021\_contig\_number\_9765SRS1041138\_contig\_number\_9332SRS1041139\_contig\_number\_35SRS1041157\_contig\_number\_5264SRS1055038\_contig\_number\_604SRS1055056\_contig\_number\_1083SRS1055067\_contig\_number\_2285SRS142980\_contig\_number\_990SRS143991\_contig\_number\_9586SRS148196\_contig\_number\_32474SRS148874\_contig\_number\_9537SRS150029\_contig\_number\_1893SRS150029\_contig\_number\_18998SRS892882\_contig\_number\_9027SRS892882\_contig\_number\_13240SRS893252\_contig\_number\_489SRS893341\_contig\_number\_3208SRS971275\_contig\_number\_222

species

SRS013382\_contig\_number\_471SRS044535\_contig\_number\_18390SRS148319\_contig\_number\_7187
3
310297

species

SRS016629\_contig\_number\_412SRS021948\_contig\_number\_31466SRS054352\_contig\_number\_8667SRS055017\_contig\_number\_19613SRS1041095\_contig\_number\_5090
5
29523

2

SRS014948\_contig\_number\_19764SRS1041164\_contig\_number\_2180
338188
species

species
2

SRS016267\_contig\_number\_8578SRS019787\_contig\_number\_9212
626929


SRS020622\_contig\_number\_444
1
2291999
species

9

SRS016629\_contig\_number\_2171SRS016954\_contig\_number\_22411SRS019445\_contig\_number\_16177SRS047433\_contig\_number\_1502SRS049446\_contig\_number\_9514SRS098073\_contig\_number\_14864SRS1055043\_contig\_number\_18592SRS143895\_contig\_number\_43792SRS149325\_contig\_number\_4346
1262754
species


SRS020394\_contig\_number\_2653SRS893230\_contig\_number\_6328
2
357276
species

species
2

SRS047044\_contig\_number\_7795SRS053214\_contig\_number\_12633
820

457387
2

SRS053335\_contig\_number\_10181SRS098073\_contig\_number\_16738
species

species
204516
27

SRS014235\_contig\_number\_2813SRS014683\_contig\_number\_14947SRS015217\_contig\_number\_20275SRS015579\_contig\_number\_9094SRS016989\_contig\_number\_8404SRS017821\_contig\_number\_10186SRS019030\_contig\_number\_11021SRS020233\_contig\_number\_37796SRS022609\_contig\_number\_41223SRS043841\_contig\_number\_10637SRS045645\_contig\_number\_9773SRS050974\_contig\_number\_contig-100\_58.24097SRS051031\_contig\_number\_30428SRS051882\_contig\_number\_10070SRS053087\_contig\_number\_992SRS053398\_contig\_number\_16752SRS101376\_contig\_number\_17389SRS103987\_contig\_number\_17627SRS1041091\_contig\_number\_20245SRS1041133\_contig\_number\_3046SRS1041138\_contig\_number\_6082SRS1041144\_contig\_number\_6778SRS1041164\_contig\_number\_1827SRS142890\_contig\_number\_23254SRS144603\_contig\_number\_11331SRS893369\_contig\_number\_11192SRS893383\_contig\_number\_26153

818
8

SRS013687\_contig\_number\_15109SRS015431\_contig\_number\_56495SRS016989\_contig\_number\_7112SRS021153\_contig\_number\_110SRS021948\_contig\_number\_231SRS022713\_contig\_number\_717SRS143085\_contig\_number\_904SRS893256\_contig\_number\_857
species

species
2
291645

SRS016095\_contig\_number\_2214SRS140492\_contig\_number\_20513

species
671267
4

SRS018656\_contig\_number\_184SRS024388\_contig\_number\_3900SRS1041132\_contig\_number\_6072SRS893327\_contig\_number\_1855


SRS893170\_contig\_number\_4100
1
291644
species

species

SRS017521\_contig\_number\_17249SRS019601\_contig\_number\_21775SRS058723\_contig\_number\_3584SRS063190\_contig\_number\_10192SRS074964\_contig\_number\_1293SRS077730\_contig\_number\_36SRS147377\_contig\_number\_2492
7
817


SRS014683\_contig\_number\_617SRS015694\_contig\_number\_15016SRS023914\_contig\_number\_9494SRS045244\_contig\_number\_777SRS063518\_contig\_number\_36532SRS148817\_contig\_number\_1161
6
47678
species

46506
17

SRS012273\_contig\_number\_31270SRS020233\_contig\_number\_9982SRS042966\_contig\_number\_18913SRS049712\_contig\_number\_18520SRS053398\_contig\_number\_3782SRS064645\_contig\_number\_94SRS101433\_contig\_number\_5460SRS1041037\_contig\_number\_9359SRS1041130\_contig\_number\_2650SRS142503\_contig\_number\_2671SRS143372\_contig\_number\_1305SRS143722\_contig\_number\_4314SRS147377\_contig\_number\_9027SRS147614\_contig\_number\_2325SRS149075\_contig\_number\_963SRS893187\_contig\_number\_11048SRS893288\_contig\_number\_436
species

171552
2
family

genus
577309
2

1
454154

SRS078176\_contig\_number\_16088
species

species
1

SRS1055022\_contig\_number\_12786
1263095
